# An Electronic Clinical Decision Support Tool to Assist Primary Care Providers in Cardiovascular Disease Risk Management: Development and Mixed Methods Evaluation

**DOI:** 10.2196/jmir.1258

**Published:** 2009-12-17

**Authors:** David P Peiris, Rohina Joshi, Ruth J Webster, Patrick Groenestein, Tim P Usherwood, Emma Heeley, Fiona M Turnbull, Alexandra Lipman, Anushka A Patel

**Affiliations:** ^2^Sydney Medical School-WesternUniversity of SydneySydneyAustralia; ^1^The George Institute for International HealthUniversity of SydneySydneyAustralia

**Keywords:** Decision support systems, clinical, cardiovascular diseases, physicians, family, Aborigines, Australian

## Abstract

**Background:**

Challenges remain in translating the well-established evidence for management of cardiovascular disease (CVD) risk into clinical practice. Although electronic clinical decision support (CDS) systems are known to improve practitioner performance, their development in Australian primary health care settings is limited.

**Objectives:**

Study aims were to (1) develop a valid CDS tool that assists Australian general practitioners (GPs) in global CVD risk management, and (2) preliminarily evaluate its acceptability to GPs as a point-of-care resource for both general and underserved populations.

**Methods:**

CVD risk estimation (based on Framingham algorithms) and risk-based management advice (using recommendations from six Australian guidelines) were programmed into a software package. Tool validation: Data from 137 patients attending a physician’s clinic were analyzed to compare the tool’s risk scores with those obtained from an independently programmed algorithm in a separate statistics package. The tool’s management advice was compared with a physician’s recommendations based on a manual review of the guidelines. Field test: The tool was then tested with 21 GPs from eight general practices and three Aboriginal Medical Services. Customized CDS-based recommendations were generated for 200 routinely attending patients (33% Aboriginal) using information extracted from the health record by a research assistant. GPs reviewed these recommendations during each consultation. Changes in CVD risk factor measurement and management were recorded. In-depth interviews with GPs were conducted.

**Results:**

Validation testing: The tool’s risk assessment algorithm correlated very highly with the independently programmed version in the separate statistics package (intraclass correlation coefficient 0.999). For management advice, there were only two cases of disagreement between the tool and the physician. Field test: GPs found 77% (153/200) of patient outputs easy to understand and agreed with screening and prescribing recommendations in 72% and 64% of outputs, respectively; 26% of patients had their CVD risk factor history updated; 73% had at least one CVD risk factor measured or tests ordered. For people assessed at high CVD risk (n = 82), 10% and 9%, respectively, had lipid-lowering and BP-lowering medications commenced or dose adjustments made, while 7% newly commenced anti-platelet medications. Three key qualitative findings emerged: (1) GPs found the tool enabled a systematic approach to care; (2) the tool greatly influenced CVD risk communication; (3) successful implementation into routine care would require integration with practice software, minimal data entry, regular revision with updated guidelines, and a self-auditing feature. There were no substantive differences in study findings for Aboriginal Medical Services GPs, and the tool was generally considered appropriate for use with Aboriginal patients.

**Conclusion:**

A fully-integrated, self-populating, and potentially Internet-based CDS tool could contribute to improved global CVD risk management in Australian primary health care. The findings from this study will inform a large-scale trial intervention.

## Introduction

 Cardiovascular disease (CVD) accounts for 18% of the total disease burden and 11.2% of health system expenditure in Australia [1]. Australian Aboriginal peoples experience around five times greater CVD burden than other Australians [2]. Despite recent gains, CVD remains Australia’s biggest killer, accounting for 46,134 deaths and disability in around 1.4 million Australians in 2005 [1]. Although effective preventive therapies are available for people at high risk of a first and subsequent CVD event [3-7], substantial challenges remain in translating this evidence into clinical practice. Our recent studies of CVD risk management in mainstream Australian general practice and indigenous health service settings found around half of routinely attending adults lacked sufficient information to comprehensively screen for CVD risk. For those identified at high CVD risk, only a minority (31% in mainstream general practice settings and 40% in indigenous health services) were prescribed guideline-indicated medications [8,9].

The reasons for suboptimal implementation of clinical guidelines include complex and multiple barriers at the health system, doctor, and patient level [10]. For a time-constrained general practitioner (GP), consolidating numerous guidelines to make clinical decisions is challenging. This is particularly true for CVD, where overall or absolute risk assessment is recommended and simultaneous management of multiple risk factors is required. Despite guideline endorsement of the absolute risk-based approach, few Australian GPs use cardiovascular risk assessment tools to guide management [11,12].

Clinical decisions support (CDS)—in Australia also commonly called electronic decision support (EDS)—is one of the most promising interventions to improve uptake of guideline-based recommendations in clinical practice. In two systematic reviews on the effectiveness of CDS, around two-thirds of studies demonstrated improvement in practitioner performance [13,14]. Key features of successful interventions included instantaneous output generation for use at the point-of-care, minimal data entry, provision of automatic prompting for GPs, and a requirement that GPs actively respond to recommendations.

A number of controlled evaluations of CDS systems that are integrated with electronic medical records (EMRs) have been conducted in the areas of CVD risk and diabetes [15-19]. They have shown variable improvements in risk factor screening/documentation and overall processes of care. Beyond trial settings, several countries have successfully implemented large-scale CDS systems for CVD risk in primary care settings. In the United Kingdom, an electronic CVD risk assessment (but not decision support) package is being integrated into one of the most commonly used GP software systems [20]. In the United States, the ATHENA decision support system is able to be integrated with a variety of primary care software platforms to promote guideline-based management of blood pressure (BP) [21]. In New Zealand (NZ), an Internet-based CVD risk management system based on the New Zealand Guidelines Group recommendations [22] has been fully integrated into the country’s most popular medical software platform EMR. This system has demonstrated improvements in uptake of CVD risk assessments [23]. Although there have been attempts in Australia to consolidate evidence about CVD management into a point-of-care paper chart tool [24], GPs would prefer decision support in an electronic format [12].

Here we outline our methods for the development of a CDS tool and present the findings of a preliminary evaluation of its use in primary care settings. This forms the first stage of a broader research and development program that will lead to the implementation and controlled evaluation of a tool that is fully integrated into Australian primary care software systems.

## Methods

### Development of the CDS Tool

For risk assessment, an algorithm was written based on the 1991 Framingham risk equation to predict 5-year risk of a first CVD event (coronary heart disease, stroke, congestive heart failure, peripheral vascular disease) [25]. Recognizing that this equation might underestimate risk for certain clinical conditions and for specific ethnic groups, adjustments were made using recommendations from the New Zealand Guidelines Group and guidelines from the 2004 National Heart Foundation (NHF) of Australia [26,27]. The risk factor variables and adjustments are summarized in Textbox 1.

**Framingham risk equation variables and adjustments used for calculation of 5-year CVD risk in the CDS tool**

**Framingham risk factor variables:**

- Age
- Sex
- Smoking status
- Blood pressure (BP)
- Total and high-density lipoprotein cholesterol levels
- Presence of diabetes
- Presence of left ventricular hypertrophy

**5% increase to the baseline risk score is made once only if any of the following are present:**

- History of premature CVD in a first-degree relative
- Body mass index ≥ 30 kg/m^2^
- Total cholesterol > 8 mmol/L
- Systolic BP > 170 mmHg
- Diastolic BP > 100 mmHg
- Diabetes duration > 10 years
- Glycosylated hemoglobin (HbA1C) > 8% for the last 12 months
- High-risk ethnic background (Aboriginal, Torres Strait Islander, Maori, Pacific peoples, South Asian)

**Age ≥** **75 years and calculated 5-year risk < 15%, then risk is adjusted to 15%**

**If the following high-risk conditions are present and calculated 5-year risk is < 20%, then risk is adjusted to 20%:**

- Established CVD (coronary artery disease, ischemic cerebrovascular disease, peripheral vascular disease)
- Left ventricular hypertrophy
- Genetic dyslipidemias
- Diabetes and chronic kidney disease (estimated glomerular filtration rate [eGFR] < 60 mL/min/1.73 m^2^)
- Proteinuria (defined as either albumin to creatinine ratio ≥ 30 mg/mmol or proteinuria > 1 g/day)

To define the risk management outputs of the tool, pharmacological treatment recommendations from six Australian CVD-related guidelines current in 2007 were consolidated into a single algorithm [26,28-31]. The thresholds and treatment targets for BP, lipid, and anti-platelet management are summarized in Textbox 2.

**Indications and target levels for CVD medication management programmed into the CDS tool**

1. **Anti-platelet medication indications:**
  - Established coronary heart disease
  - Diabetes
  - Ischemic cerebrovascular disease
2. **BP medication**
  - **Indications for commencing treatment:**
    - BP > 125/75 mmHg for the following:
      - Diabetes and proteinuria (defined as either albumin to creatinine ratio > 30 mg/mmol or proteinuria > 1 g/day)
      - Diabetes and chronic kidney disease (defined as eGFR < 60 mL/min/1.73 m^2^)
    - BP > 130/80 mmHg for all others with diabetes or isolated proteinuria
    - BP > 140/90 mmHg and any one of the following:
      - Established CVD
      - Chronic kidney disease (eGFR < 60 mL/min/1.73 m^2^)
      - Aboriginal, Torres Strait Islander, Pacific Islander, Maori, South Asian background
      - Adjusted 5-year CVD risk > 10% (assuming lifestyle advice given for 3-6 months)
    - BP > 150/95 mmHg and adjusted 5-year CVD risk < 10% (assuming lifestyle advice given for 3-6 months)
  - **Target treatment levels:**
    -
      - BP < 125/75 mmHg for those with diabetes and proteinuria
      - BP < 130/85 mmHg for:
        - All others with diabetes
        - Chronic kidney disease
        - Isolated proteinuria
        - Age < 65 years
      - < 140/90 mmHg for all others
3. **Lipid medication**
  1. **Indications for commencing treatment:**
    - Established CVD at any level
    - Genetic lipid disorders at any level
    - Diabetes and serum triglycerides > 2 mmol/L
    - Low-density lipoprotein cholesterol > 2.5 mmol/L and any of the following:
      - Diabetes
      - Aboriginal or Torres Strait Islander
      - Adjusted 5-year CVD risk > 15%
  2. **Target treatment levels:**
    - Low-density lipoprotein cholesterol < 2.5 mmol/L

**Figure 1:**
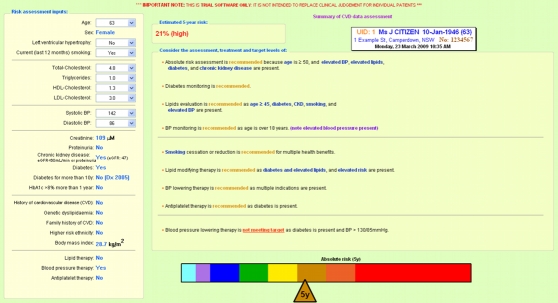
Sample CDS output with complete information and color bar

**Figure 2:**
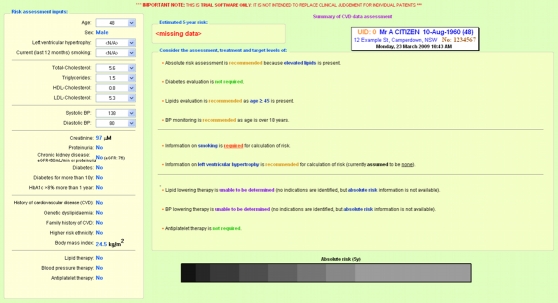
Sample CDS output with incomplete information and greyscale bar

The risk assessment and management algorithms were programmed into a stand-alone software package (Igor Pro 6, WaveMetrics Inc, Portland, OR, USA) that produced a single-page output. If there was complete risk factor information available, a risk score was generated and plotted along a color spectrum bar and treatment recommendations were provided. If information required for absolute risk assessment was absent, the output identified the variables missing and the color bar was changed to greyscale. Because many Australian guidelines are not exclusively risk based, some treatment recommendations could still be made despite incomplete risk factor information. Examples of these two types of output are shown in Figure 1 and Figure 2.

### Validation Testing of the Tool

De-identified data from all consecutive patients with complete risk factor information attending a specialist vascular clinic over a 3-month period (May to August 2008, n = 137) were entered into the tool by a trained research assistant to generate CDS outputs. The validity of these outputs was assessed in two parts. First, a researcher who was not involved with the algorithm development programmed the Framingham risk equation and adjustments into a second statistical software package, STATA version 9.2 (Stata Corporation, College Station, TX, USA). Correlation between risk scores generated from the CDS tool and the STATA program were assessed. Second, an experienced physician, blinded to the CDS tool management recommendations, reviewed the risk assessment data for each patient. She then performed a manual review of the guidelines and assessed whether anti-platelet, BP-lowering, and lipid-lowering medications were indicated or whether targets were being met for those patients already prescribed BP-lowering and lipid-lowering drugs. Agreement between the CDS tool and the physician’s recommendations was assessed.

### Field Testing in Primary Health Care

**Figure 3:**
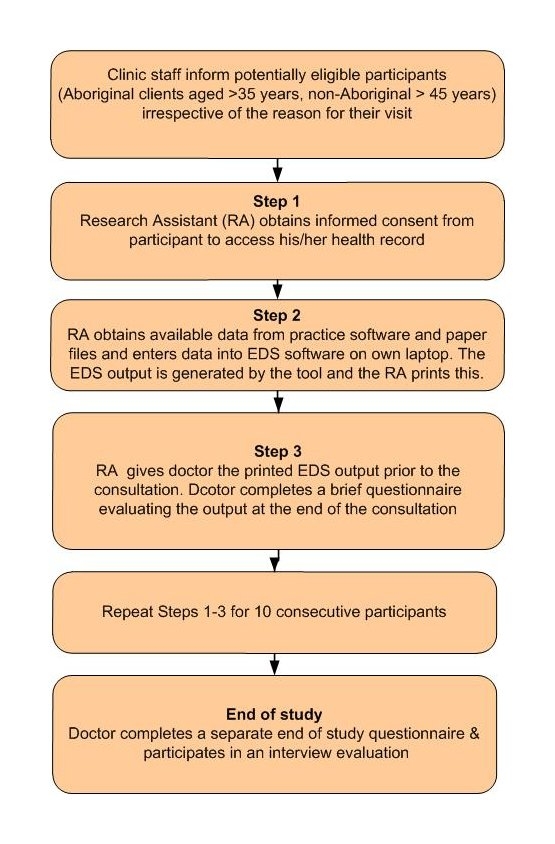
Study schema

The tool was field tested in two different Australian primary health care settings: eight teaching general practices in Sydney and three Aboriginal Medical Services (AMSs) in New South Wales. Sampling was purposive and sought GPs interested in research and training who might critically appraise the tool and provide recommendations for its future development. A diversity sample in terms of GP age, gender, and size of practice was sought. Consecutive, routinely attending patients (Aboriginal ≥ 35 years, non-Aboriginal ≥ 45 years) were invited from the waiting room to participate. The patient age range is based on Australian guideline recommendations for absolute risk assessment screening [32]. Each GP had outputs generated for around 10 patients. This number was considered sufficient to allow (1) adequate exposure to a variety of tool outputs, (2) an appreciation of the tool’s application in a typical working day, and (3) minimal administrative burden to the GP or the practice. Figure 3 provides a schema for how the study was conducted. Because the pilot version of the tool was built using stand-alone software, it lacked the ability to pre-populate with demographic and clinical data already existing in the EMR. Thus, the key role of the research assistant was to act as a proxy for this pre-populating feature by accessing the relevant risk factor information from the patient’s EMR. In essence, this simulated the situation that might occur if the tool was built into the GP’s practice software system. The resultant output was given to GPs prior to the consultation for review with their patients.

### Evaluation and Analyses

A mixed methods evaluation was conducted following the methods outlined by Tashakkori and Teddlie [33]. Specifically, the quantitative and qualitative components were equally weighted and combined simultaneously to obtain an understanding of the effectiveness (quantitative), acceptability (quantitative and qualitative), and feasibility (qualitative) of a CDS tool for CVD risk management in primary care settings.

At the end of each consultation, GPs completed a short survey on their attitudes about the tool and management provided. At study completion, GPs completed a second survey on their practice characteristics. This survey adapted some questions from a previously published instrument [34]. GPs then participated in an in-depth interview evaluation. Interviews were semistructured and conducted by a GP researcher who had a practical working knowledge of the tool in clinical settings. Interviews covered three domains: (1) general attitudes about the tool and its impact on the consultation; (2) a review of specific tool outputs; (3) recommendations for future tool development. Full details of the survey instruments and interview guide are provided in Multimedia Appendix 1-3.

Descriptive statistics and quantitative analyses were conducted using SAS version 9.1 (SAS Institute Inc, Cary, NC, USA). Management decisions were assessed as to whether GPs acted on recommendations from the tool output. Interview recordings were professionally transcribed, and thematic content analysis was performed drawing on the methods outlined by Patton [35]. Interview transcripts were initially reviewed in their entirety to become familiar with the data. They were then open coded to core thematic categories and these analyses were conducted contemporaneously with data collection. At the end of study, the investigator team met on several occasions to determine how these open-coded categories would be relationally grouped to determine the final major themes. NVivo 8 (QSR International, Melbourne, Victoria, Australia) was used to help organize the data through this analysis process.

The study was approved by both the Sydney South West Area Health Service and Aboriginal Health and Medical Research Council ethics committees. Patients and GPs gave written informed consent to participate in the study. Signed agreements were obtained from the three participating AMSs.

## Results

### Validation of the Tool

The tool’s risk assessment algorithm showed near perfect correlation with the independently programmed algorithm used in STATA (intraclass correlation coefficient 0.999). The variation was wholly explained by different rounding methods used in each software program. For prescribing recommendations, agreement between the tool and the physician’s recommendations for initiation of anti-platelet and lipid treatment was 100%. Agreement on meeting guideline targets for those already prescribed BP- and lipid-lowering treatments was also 100%. Agreement on initiation of BP treatment was 97% (kappa 0.95). In both cases of disagreement, the BP was < 125/75 mmHg and the physician judged that treatment was not indicated, while the tool recommended that treatment could not be determined due to missing information on proteinuria.

### Field Testing – Quantitative Evaluation

Twenty-one GPs participated in the study. Practices varied greatly in size, ranging from a solo GP practice with minimal administrative support to a large practice with 23 GPs and 15 nurses. Table 1 outlines GP characteristics and their use of electronic practice management features. Table 2 shows the risk factor characteristics of the patient population by Aboriginal status and prescribing rates of preventive CVD medications.

**Table 1:** Characteristics of the 21 participating GPs

	No.	%
**Male**	12	57
**Age group** (years)		
20-29	1	5
30-39	3	14
40-49	11	52
50+	6	29
**Postgraduate qualifications**		
Fellowship of the Royal Australian College of GPs	15	71
Diploma (eg, obstetrics, child health)	11	52
Master (eg, public health)	4	19
**Participate in research sometimes or often**	19	90
**Use of Internet at least once daily**	19	90
**Electronic practice software features always used**		
Medication prescribing	20	95
Automated pathology results downloaded	19	90
Online billing	14	67
Electronic patient recalls	13	62
Scanning of paper documents	12	57
Electronic care plans	12	57
Disease registers	7	33
**Frequency of performing cardiovascular risk assessments for Aboriginal 35+ years, non-Aboriginal 45+ years**		
Never	3	14
Less than 50% of the time	16	76
Greater than 50% of the time	2	10
**Preferred method of assessing risk**		
New Zealand guidelines color charts	15	71
Calculators within medical software	2	10
Other methods (eg, downloaded calculator)	1	5
Risk assessment never performed	3	14

**Table 2:** Baseline risk assessment characteristics of 200 patients attending their GP^a^

	Non-Aboriginal (n = 134)	Aboriginal (n = 66)	Total (n = 200)
**Age in years** (mean ± SD)	51.5 ± 29.8	50.1 ± 10.62	51.1 ± 25.1
**Female**	79 (59%)	45 (68%)	124 (62%)
**Recorded diabetes**	37 (28%)	30 (46%)	68 (34%)
**Current smoker^b^**	36 (27%)	33 (50%)	69 (35%)
**5-year adjusted CVD risk**			
Low risk (< 10%)	28 (21%)	16 (24%)	44 (22%)
Moderate risk (10-15%)	12 (9%)	9 (14%)	21 (11%)
High risk (> 15%), excluding established CVD	28 (21%)	11 (17%)	39 (20%)
Established CVD	30 (22%)	13 (20%)	43 (22%)
Unable to estimate risk due to missing information	36 (27%)	17 (26%)	53 (27%)
**Medication prescribed**			
Lipid-lowering	67 (50%)	31 (47%)	98 (49%)
Anti-platelet	50 (37%)	20 (30%)	70 (35%)
BP-lowering	85 (63%)	37 (56%)	122 (61%)

^a^ Reported as no. (%) unless otherwise indicated. Percentages may not add to 100% due to rounding.

^b^ Current smoker or quit within past 12 months.

**Figure 4:**
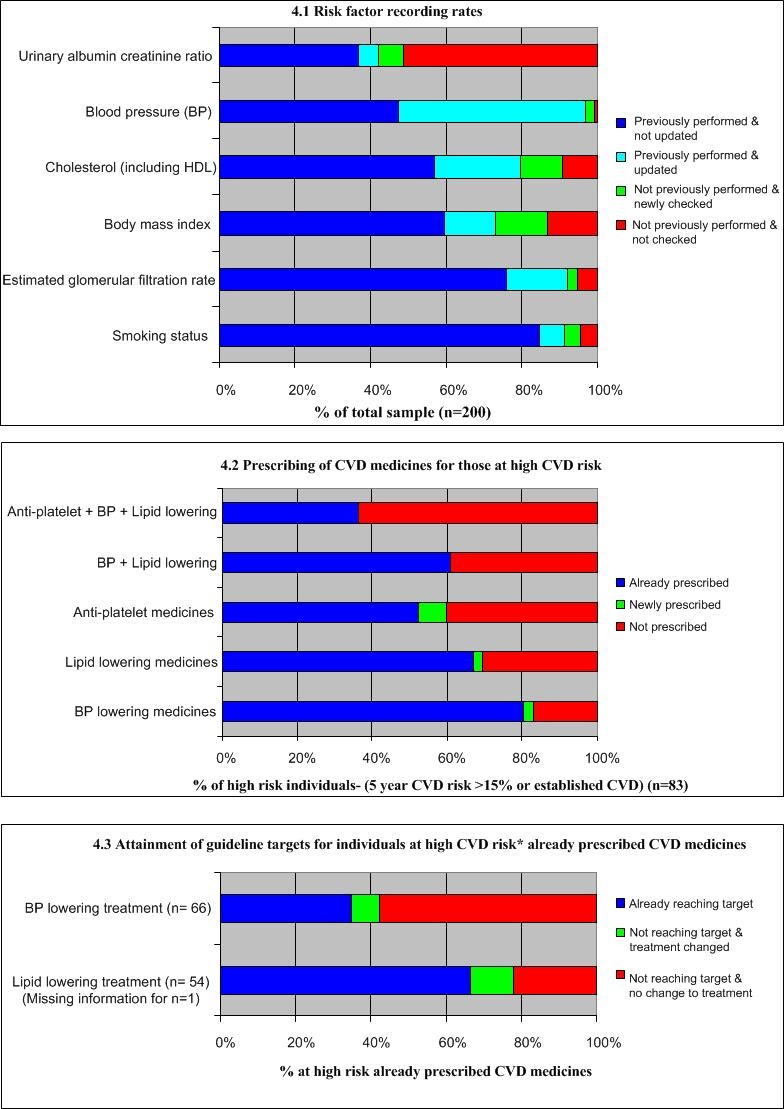
CVD management practices before and after a consultation involving the CDS tool

For the 200 CDS outputs generated for review, GPs agreed or strongly agreed that the output was easy to understand (77% of outputs), that screening and prescribing recommendations were appropriate (72% and 64% of outputs, respectively), and that recommendations on treatment targets were appropriate (70% of outputs). Fifty-two (26%) patient records were updated with CVD-related information, most commonly family history, past history of CVD, and smoking status. Figure 4 highlights the changes in risk factor screening and management following the consultation. Ninety-five (48%) patients received changes to their management, of whom 49 (52%) received lifestyle advice on CVD risk factors. For people assessed at high CVD risk (n = 82), 10% and 9%, respectively, had lipid-lowering and BP-lowering medications commenced or dose adjustments made, while 7% newly commenced anti-platelet therapy.

### Field Testing – Qualitative Evaluation

All GPs participated in the interview evaluation, with interviews ranging from approximately 30 to 60 minutes duration. One interview was conducted with a pair of participants, two interviews were conducted over the telephone, and the remainder were individual face-to-face interviews. Three major themes arose from the interview content analysis that will be reported here. A fourth substantive theme was identified that related to how tools are used in general practice and the role of evidence-based medicine in decision making. As this issue extends beyond factors related to the CDS tool and was not a specific objective of the study*,* an in-depth analysis of this theme will be conducted separately.

#### Theme 1: Systematic Provision of Care

Most GPs felt that the tool was effective in providing comprehensive support in CVD risk management, both at the point-of-care and as an adjunct to reviewing their clinical performance.

Interview 7: Male GP over 60 years Oh well it does help, because it’s your data there...and you look at it and you think “Oh gee, that’s not there. I haven’t put that in” or “Well yeah, they are not to target there”.... So it’s just a reminder that you might think you’re doing okay, but there’s nothing like seeing the actual figures to make you realize that “Okay, there’s room for improvement here.”

Interview 12: Male GP 40-49 years I think it was quite a good thing because you would finish the consultation about whatever that was about and then you’d almost have a separate time set for looking at cardiovascular risk.... Otherwise, I would think about doing it through the consultation, but you just seem to forget and then you would think “Oh damn it, I should have done that.” So having that piece of paper there gave you that conversation: “Well now we’ve finished everything, let’s look at this.”

Interview 19: Male AMS GP 40-49 years I think it’s useful to us.... It’s basically like a mini audit. So anything that makes you look a little bit deeper at the person sitting in front of you is always worthwhile....

Importantly, however, recommendations based on single risk factor readings, out-of-date, or even false readings undermined the full benefit of such a tool. GPs sought clarification on the underlying assumptions in how risk was calculated and management recommendations were made. For the few GPs who were dissatisfied with the tool’s recommendations, these issues accounted for much of that dissatisfaction.

Interview 11: Male GP 50-59 years It gives information which, as it’s blandly presented, you go, “How did you get that?...” I got a couple of people where I got a 20% number and you go, “Oh that’s madness, that’s not you,” and often because it’s based on single digit information…like a single blood pressure.

Interview 17: Female AMS GP 20-29 years The other issue I have with this data which came up is it uses the last available input.... I think what would be really good is something that came up and said, “This is the risk, but we’ve used data that’s three years out of date.... You need to be doing it again.”... just a reminder to say, “Ah, I should be thinking about that for everyone.” I think that would be really useful.

GPs further highlighted the need for ongoing revision as guidelines are updated.

Interview 11: Male GP 50-59 years We’re used to every month getting a download of the new drug file, the new program data…with therapeutic guidelines.... There’s a little button that says, this is emerging guidelines or these are the things that have just been incorporated within it.... You don’t really want to be working on guidelines that are too old....

#### Theme 2: Risk Communication

Despite only brief exposure to the tool, many GPs commented on its role in risk communication. The synthesis of multiple risk factor information onto a single page appeared to promote a beneficial dialogue with patients. The need for an evaluation from the patient perspective was highlighted.

Interview 2: Female AMS GP 40-49 years I think the biggest impact is that it changed the way I talked about what I was doing with patients, in that it made it a much more slick, neat package to describe the normal screening that you do for risk management. And so I felt it was easier to deliver some description of where they’re at now. And from their point of view, I mean it’s hard to know, but they seemed to understand that it was a multifactorial thing, rather than just being one of those single disease problems.... The thing that I don’t really know, that I guess would be useful, is what they think when they walk out the door, what they actually understand of what I’ve said.

Most noteworthy was the prominence of the color bar (see Figure 1) in promoting discussions about risk management.

Interview 15: Female AMS GP 30-39 years I like this one [referring to the color bar].... I mean, everyone knows that red means danger, so if they’re heading towards this one, it’s a lot more visual, the impact....

Interview 9: Female GP 50-59 years I could see the potential for using it to discuss with the patient.... I like the fact that it had that nice bar with the color gradations because my other previous use of trying to describe risk has been using that one from the New Zealand calculator, and it’s very complicated. It’s too complicated. And I find it really, you know, very pretty, but difficult for the patient to really get much sense out of. So I liked that single bar. I thought that was much more useful for people.

Interview 4: Male AMS GP 30-39 years Yeah, and even the colored diagram is really helpful in seeing and being able to say, “…Look, this is going into orange – this says high in red.” And there’s almost an emotional response to the colors that come back there that is actually really useful compared to me saying, “Look, people with diabetes have heart attacks and strokes.”

Additionally, some GPs considered that interactively changing the risk factor profile and resulting risk score (including color category) would facilitate conversations about the relative contributions of individual risk factors to overall risk.

Interview 8: Male GP 30-39 years I could think on the absolute risk bar, if you’ve got an arrow for where they sit now, potentially you could have an arrow for if you were to modify what was modifiable and where could you get.... “You [the patient] could ultimately work your way down to here,” and it might be a way of saying, “Well, there is the gap,” and that might be helpful as a motivator.

Interview 16: Male AMS GP 50-59 years So that gets me thinking about talking to the patient about the relative merits of putting them on drugs compared to smoking, and I think as an interactive thing I could bring up this thing and change her smoking or change her BMI...and say, “This is a much simpler way of dramatically changing your absolute risk.”

#### Theme 3: Challenges for Implementation in Routine Care

While GPs felt that it was appropriate and feasible to incorporate CVD risk management into routine care, the time pressures in doing so were highlighted. A major potential constraint identified would be the time required for data entry. A common view expressed was that a tool integrated with practice software would need to be pre-populated with as much risk factor information as possible.

Interview 4: Male AMS GP 30-39 years I think it depends on the patient. The ones where I think it takes most time are those where it’s not been brought up and it turns out that the risk is high. So where you feel the stakes are higher...and it’s not really been on your radar and it’s certainly not been on the patient’s radar. There aren’t that many of those. For most of the patients where the risk is high, you’re already aware that their risk is high.... In that context, it isn’t that much extra work....

Interview 10: Male GP 50-59 years I’m not sure how you can do it, because some are from pathology reports coming back, some things you have to measure, and then some people don’t put it in the right boxes. They just type in. So if you don’t put it in the right place, then the software won’t be able to pick it up. If I have to go enter [data] into this thing, then I’m pretty sure very few people are going to do it...just like the New Zealand one.... But, if you could extract it automatically, or maybe I fill in the occasional one...then that’s fine.

Interview 11: Male GP 50-59 years One of my rules in general practice is “every 30 seconds counts,” so if it becomes something that slows the program down, if it becomes something that blocks your progress on doing what you want to do...they’re the things that would make it less usable...rather than becoming distracted by this thing because you are stuck with closing boxes and pop-ups and forced to put data in.... What I like about this [the CDS tool], it pulls information together for you so you don’t have to look through 7, 8 different places....

This was considered particularly germane to GPs who are less comfortable with EMR use and where information may not be stored in an extractable format.

Interview 8: Male GP 30-39 years Less-computer-literate doctors will find it less useful because they don’t have the information there.... So, if people put garbage in, you will get garbage out, and I don’t think that is going to change..... I can’t imagine a paper file doctor wanting to use the tool in the first place. So I think your target is only likely to be people who are more computer savvy.

Some GPs advised of the need for a more graphically oriented layout and innovative prompting mechanisms that avoid contributing to the already congested number of “pop-up” prompts present in their systems. Additionally, some GPs felt that the screening (as opposed to management) recommendations offered little additional value and, in their time-poor context, may distract from the recommendations about indicated preventive therapies.

Interview 2: Female AMS GP 40-49 years I find it all too wordy.... I can’t read those words while I’m sitting there with a patient. I still have to sit there and think, “What does that sentence actually mean?...” So, it needs to be very graphic, where it says the same thing to you graphically.

Interview 14: Female GP 40-49 years [The tool was] almost too busy.... I’ve only got a minute to glance at it.... People normally wait about four, six weeks to come and see me, and so they’ve got a lot of stuff they want to see me about.... I don’t need to know that lipids evaluation is recommended for those aged over 50. What you want is the real necessary stuff...those first four things (the screening recommendations) actually weren’t necessary.... You’ve got 15 minutes at most and...if you don’t have that information in the first two lines, people won’t read it.

## Discussion

This preliminary evaluation demonstrates that a valid decision support tool for CVD risk management can be successfully developed and that such a tool was favorably received by GPs working in two distinct primary health care settings. The baseline prescribing patterns of CVD medications to high-risk individuals were broadly similar to those reported in our previous Australian audit studies [8,9]. The improvements in risk factor screening and the intensification of existing therapies were promising signs of the tool’s ability to promote absolute risk-based care. It was also encouraging that despite, or perhaps because of, the high rates of Aboriginal CVD disease burden, the tool was viewed positively by AMS care providers. A large-scale controlled evaluation would clearly be needed to substantiate these preliminary study findings.

The evaluation identified key aspects of both the tool’s scientific design and functionality that are likely to be crucial for successful wider implementation. Our findings support the systematic review evidence that CDS tool features associated with improved performance include factors such as integration with routine workflow, provision of automated decision support, and provision of recommendations rather than simply assessments [14]. Perhaps the most fundamental finding from this study is that CDS tools need to be effectively incorporated into routine care and avoid being viewed as an optional, additional burden to the workload. Integration within existing medical software systems and maximal use of information contained in other parts of the EMR would reduce data entry and increase the tool’s use. Although the uptake of EMRs in the Australian primary care system is widespread for prescribing medications and pathology services, their routine use for other purposes is more variable [36]. This poses both challenges and opportunities for CDS tools. In this pilot, the research assistant accessed health information from disparate parts of the EMR, including free-text information. The ability to automatically “push” data into a CDS tool and limit burdensome data entry is dependent on the extent to which information exists in an extractable format. If the amount of extractable information is scant, this could pose a major barrier to use of CDS tools. The tool itself, however, can be utilized as a strategy to overcome this problem. If the information that is entered directly into the tool can be “pulled” back into the appropriate parts of the EMR, then there is a dual purpose being served—that of performing a clinically relevant task at the point-of-care and a data cleaning process. In practical terms, this would mean that the CDS output would either be automatically generated based on existing data or prompt the practitioner for any missing data. This missing data could then be entered directly into the tool and written back to the appropriate part of the health record, avoiding the need for double data entry. This makes future risk assessments easier to perform, affords extraction of more reliable data for auditing and quality improvement purposes, and supports the use of shared electronic health records across multiple service providers. Full EMR integration is also a key consideration in supporting other components of chronic disease management such as chronic care plans, well person’s health assessments, and audit cycles of care (all of which attract Australian government–funded rebates). This could ensure that the tool facilitates existing care, rather than competes with it.

The NZ Web-based decision support system for CVD risk has been purposefully designed to be “agnostic” to the EMR environment and is capable of pushing and pulling data with a variety of commercial products. As a centrally deployed system, there is also a mechanism for rapid implementation of updates as subsequent guidelines evolve (already a priority issue in Australia given that three new CVD-related guidelines have been released since initial programming of this tool). In order to meet these specification requirements in the Australian context, adequate resourcing and a close collaboration between researchers and EMR vendors are needed. The Medical Software Industry of Australia, which is the peak representative body for all EMR providers, the Australian Health Information Council, and the Australian government’s National E-Health Transition Authority are key stakeholders that can assist with establishing industry standards on CDS tools. Furthermore, endorsement of these tools by the peak national bodies responsible for generating and disseminating guidelines could further increase GP confidence in their validity.

An important consideration for future development of the tool is to more fully understand its impact on communication of CVD risk between care provider and patient. This study confirms previous findings that GPs use these tools to facilitate the provider–patient interaction [12]. Of particular note was the role of the color spectrum bar in communicating risk information and the desire to interactively change this based on different risk scenarios. While this tool examined decision support for the care provider, further work examining how best to provide decision support for the patient is needed. This includes identifying acceptable formats for conveying risk information, evaluating the impact of decision support on health care interactions, and exploring its potential for use outside the clinical consultation (eg, self-management programs and personal eHealth records).

### Limitations

A limitation of this preliminary evaluation was that changes in care provider practices were based on a single consultation, reducing the ability to assess the potential impact of the CDS tool over time. A second potential limitation was the sampling method. Rather than seek a representative sample, we sought GPs who might actively contribute to the future development of the tool. AMSs were considered important settings to assess whether the tool was acceptable for use in a population with high levels of health disadvantage. Despite this purposive sampling, the types of medical software used, the electronic features used within those software systems, and the rates of performing absolute risk assessments were broadly similar to those reported in the Australian literature [12,36].

### Future Implications

The implications of a CDS tool for CVD risk management extend well beyond being a point-of-care clinical resource. Data from UK CVD risk programs have allowed for the generation of population-specific risk prediction equations that outperform Framingham-based algorithms [20]. The NZ decision support system, combined with linkage to mortality and hospital databases, is similarly allowing for rapid advances in CVD risk factor epidemiology. The combination of a centrally managed Internet-based system with local management of program specifics by primary health organizations allows for a “ground up” approach to incorporating population health aspects into such systems. Along with epidemiological advances, both the UK and NZ systems allow for the use of large-scale primary care data to monitor health system performance. In Australia, such systems will play an integral role in the broader eHealth strategies being proposed to reform the health care system [37-39]. Performance measures in CVD risk management are integral to the UK Quality and Outcomes Framework and are allowing for large-scale analyses of regional variation and progress in reducing health inequalities [40]. In Australia, this is especially pertinent to addressing Aboriginal health inequities where specific indicators for the measurement and reduction of CVD risk are proposed [41]. Awareness of these broader issues and incorporation of the major study findings into the next phase of the project will provide a strong foundation to develop, implement, and evaluate an integrated CVD risk management system in Australian primary health care.

## Multimedia Appendix 1

GP questionnaire completed at the end of each patient consultation

## Multimedia Appendix 2

GP questionnaire completed at the end of the study

## Multimedia Appendix 3

GP interview guide

## Abbreviations

AMS: Aboriginal Medical Service
BP: blood pressure
CDS: clinical decision support
CVD: cardiovascular diseaseeGFR: estimated glomerular filtration rate
EMR: electronic medical record
GP: general practitioner
NZ: New Zealand
