# Supplementary material for: An Electronic Clinical Decision Support Tool to Assist Primary Care Providers in Cardiovascular Disease Risk Management: Development and Mixed Methods Evaluation
Source: J Med Internet Res. 2009 Dec 17;11(4):e51. doi: 10.2196/jmir.1258 (PMC2802562; doi:10.2196/jmir.1258)
Supplement: Supplementary file 1 [file jmir_v11i4e51_app1.pdf]

Department of General Practice, Westmead Hospital

Electronic Decision Support Feasibility Study
End of consultation questions

THE GEORGE INSTITUTE for International Health

Patient ID: I__I__I__I__I__I__I Sex (F/M): I__I

We are collecting this information to evaluate the EDS tool and develop it further. This is an untested tool; please use your clinical judgment to manage your patient. Your assistance is appreciated.

Please note that the questions below apply for this patient only

	**Please indicate whether you agree or disagree with the statement below**	Strongly disagree	Disagree	Neutral	Agree	Strongly agree	Don't know or not applicable
1.	The EDS printout was easy to understand	☐	☐	☐	☐	☐	☐
	The screening/ monitoring recommendations were appropriate	☐	☐	☐	☐	☐	☐
	The treatment recommendations were appropriate	☐	☐	☐	☐	☐	☐
	For patients already taking BP or cholesterol medicines, the recommendations on meeting targets were appropriate	☐	☐	☐	☐	☐	☐

2. Did you update your clinical records based on the recommendations of the EDS tool?
If yes, which of the following did you add or update?

Yes ☐ No ☐

- ☐ Family history of cardiovascular disease
- ☐ Past history of cardiovascular disease
- ☐ Smoking status
- ☐ Genetic dyslipidaemia/ Familial Hypercholesterolaemia
- ☐ Diabetes status
- ☐ Other cardiovascular disease related information (please specify):

3. Did you order or perform any of the following for your patient in this consultation?
If yes, please tick as many as apply.

Yes ☐ No ☐

☐ Blood pressure	☐ Total cholesterol	☐ Fasting Blood glucose/GTT
☐ Height & weight	☐ HDL	☐ Serum creatinine/ eGFR
☐ Waist circumference	☐ LDL	☐ Urinary Albumin Creatinine Ratio
☐ Urinalysis	☐ Triglycerides	☐ HbA1c
☐ ECG	☐ Electrolytes	
☐ Other tests (please specify):		

4. Did you change the treatment plan for your patient?
If yes, which of the following did you change or add?

Yes ☐ No ☐

- ☐ Blood pressure lowering therapy
- ☐ Blood glucose lowering therapy
- ☐ Lipid lowering therapy
- ☐ Anti-platelet therapy (aspirin, clopidogrel, dipyrimadole etc.)
- ☐ Lifestyle modification advice (either Smoking, Nutritional, Alcohol or Physical Activity advice)
- ☐ Other treatments (please specify)
