# Supplementary material for: An Electronic Clinical Decision Support Tool to Assist Primary Care Providers in Cardiovascular Disease Risk Management: Development and Mixed Methods Evaluation
Source: J Med Internet Res. 2009 Dec 17;11(4):e51. doi: 10.2196/jmir.1258 (PMC2802562; doi:10.2196/jmir.1258)
Supplement: Supplementary file 2 [file jmir_v11i4e51_app2.pdf]

Department of General Practice, Westmead Hospital

Electronic Decision Support Feasibility Study
End of study GP questionnaire

THE GEORGE INSTITUTE *for International Health*

We are collecting this information to evaluate the Electronic Decision Support tool and develop it further. Because this is an untested tool we are interested to know your opinions about its feasibility for use in General Practice. Your responses to the questions below will give us important background information about you and your practice and will take about 15 minutes to complete. All responses are private and confidential. Your assistance is greatly appreciated.

Section I: Your Background

1.1 What is your age?

- ☐ 20-29
- ☐ 30-39
- ☐ 40-49
- ☐ 50-59
- ☐ 60 or over

1.2 What is your gender?

- ☐ Male
- ☐ Female

1.3	What is the primary language you speak at home?	
1.4	What country were you born in?	
1.5	From which university did you obtain your medical degree?	
		Year graduated:

1.6 Please list up to four of your post-graduate medical qualifications below:

Qualification	Institution	Year awarded

1.7 Are you vocationally registered?

- ☐ Yes
- ☐ No

1.8	How many sessions per week do you work at this practice?	
1.9	How many sessions per week do you work elsewhere?	

		Never	Sometimes	Often	Very often
1.10	How often do you participate in research?	☐	☐	☐	☐
1.11	How often do you conduct your own research?	☐	☐	☐	☐

Section 2: Practice Characteristics

2.1	How many of each category of the following staff are employed in this practice? *(if none then please write '0')*:	Doctors ☐<br>Nurses ☐<br>Aboriginal Health Workers ☐<br>Practice Managers ☐<br>Other administrative staff ☐<br>Allied Health professionals ☐
2.2	Which of the following best describes access to bulk-billing at your practice? *(please choose one)*	Exclusively bulk-billing ☐<br>Selective bulk-billing (eg. children, seniors, concession card holders) ☐<br>No bulk-billing ☐<br>Other billing arrangements *(please specify):*

2.3 Is your practice accredited?

☐ Yes

☐ No

If so what year was the practice first accredited? (please leave blank if you don't know): ____________

2.4 Please indicate your agreement or disagreement with the following statements about your practice.

	Strongly Disagree	Disagree	Neutral	Agree	Strongly agree
I consider this practice to be innovative.	☐	☐	☐	☐	☐
We are actively doing things to improve quality of care.	☐	☐	☐	☐	☐
After we make changes to improve quality, we evaluate their effectiveness.	☐	☐	☐	☐	☐
We have quality problems in our practice.	☐	☐	☐	☐	☐
Our procedures and systems are good at preventing errors from occurring.	☐	☐	☐	☐	☐

Section 3: Use of Information Technology

3.1	How often do you use the Internet for personal and/or professional use, including e-mail from home, work, or another location?	Several times a day ☐<br>Daily ☐<br>Weekly ☐<br>Monthly ☐<br>Less than monthly or not at all ☐
3.2	Which practice software system do you currently use at your practice?	Medical Director ☐<br>MedTech ☐<br>Best Practice ☐<br>Practix ☐<br>Other *(please specify):*

		Very Unsatisfied	Unsatisfied	Neutral	Satisfied	Very Satisfied
3.3	Overall how satisfied are you with the computer systems at your practice?	☐	☐	☐	☐	☐

3.4 Please indicate which of the following features you use in your practice.

	Not available at this practice	Available but I do not use it	I use *some* of the time	I use *most or all* of the time
Electronic medication prescribing	☐	☐	☐	☐
Electronic pathology ordering	☐	☐	☐	☐
Electronic downloads of pathology results	☐	☐	☐	☐
Electronic care plans	☐	☐	☐	☐
Electronic disease registers (eg. diabetes)	☐	☐	☐	☐
Electronically generated recalls (e.g. immunizations, pap smears)	☐	☐	☐	☐
Electronic on-line billing	☐	☐	☐	☐
Scanning of paper documents into practice software (eg. specialist letters)	☐	☐	☐	☐

3.5	How much of a barrier is each of the following to successful implementation of computer systems at your practice?			
		Not a barrier	Minor barrier	Major barrier
	Staff training	☐	☐	☐
	Privacy/ Security concerns	☐	☐	☐
	Medical software limitations	☐	☐	☐
	Technical limitations (e.g. slow response time of computers, poor technical support	☐	☐	☐

3.6	Please indicate how positive the impact of computer systems has been for each of the areas below.					
		Very negative	Somewhat negative	No effect	Somewhat positive	Very positive
	The practice of evidence based medicine	☐	☐	☐	☐	☐
	Patient-doctor communication	☐	☐	☐	☐	☐
	Patient privacy	☐	☐	☐	☐	☐
	Practice cost efficiencies	☐	☐	☐	☐	☐
	Overall patient safety (eg. reduction in medication errors)	☐	☐	☐	☐	☐

Section 4: Access to medical information

4.1	Please indicate how influential the following sources of medical information are in your practice.			
		Not influential	Somewhat influential	Very influential
	Observation and discussion with GP colleagues	☐	☐	☐
	Correspondence with specialists	☐	☐	☐
	Pharmaceutical company representatives	☐	☐	☐
	Drug product information within clinical software (eg MIMS)	☐	☐	☐
	Continuing Medical Education (CME) events	☐	☐	☐
	Conferences	☐	☐	☐
	Australian Medicines Handbook	☐	☐	☐
	Medical newspapers (eg. Medical Observer/ Australian Doctor)	☐	☐	☐
	Australian Family Physician	☐	☐	☐
	Peer-reviewed journals	☐	☐	☐
	Evidence Based Medicine guides (eg. Up to Date)	☐	☐	☐
	Personal internet searches (Google, PubMed etc.)	☐	☐	☐
	Clinical guidelines from professional organisations	☐	☐	☐

4.2	Please indicate how influential the following clinical guidelines are on your clinical practice.				
		I am not aware of this guideline	Not influential	Somewhat influential	Very influential
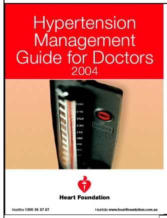	National Heart Foundation "Hypertension Management Guide for Doctors"	☐	☐	☐	☐
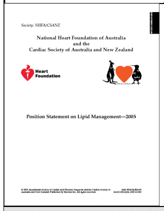	National Heart Foundation and Cardiac Society of Australia and New Zealand "Position Statement on Lipid Management"	☐	☐	☐	☐
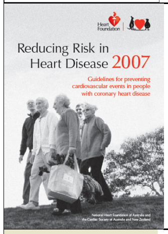	National Heart Foundation "Reducing Risk in Heart Disease"	☐	☐	☐	☐
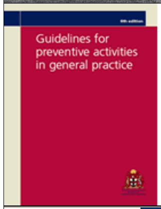	The RACGP "Red Book"- "Guidelines for Preventive activities in General Practice"	☐	☐	☐	☐
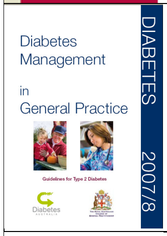	Diabetes Australia and RACGP "Diabetes Management in General Practice"	☐	☐	☐	☐
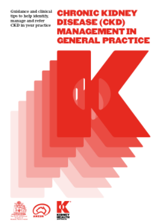	Kidney Health Australia- "Chronic Kidney Disease Management in General Practice"	☐	☐	☐	☐
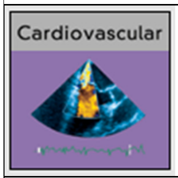	Therapeutic Guidelines- Cardiovascular	☐	☐	☐	☐
	The Pharmaceutical Benefits Scheme criteria for lipid lowering therapies	☐	☐	☐	☐

Section 5: Use of cardiovascular risk assessments

	For patients over 45 years (or over 35 years for indigenous patients), on average how often would you calculate cardiovascular risk?			
	Always ☐	More than 50% of the time ☐	Less than 50% of time ☐	Never ☐
	If you do calculate your patients' cardiovascular risk which of the following resources do you use?	Paper colour charts (eg. NPS, Heart Foundation, New Zealand) ☐		
		Risk calculators within your medical software ☐		
		On-line or downloaded risk calculators ☐		
		Other risk calculation methods: *(please specify)*		

Thank you for your participation!
