# Supplementary material for: An Electronic Clinical Decision Support Tool to Assist Primary Care Providers in Cardiovascular Disease Risk Management: Development and Mixed Methods Evaluation
Source: J Med Internet Res. 2009 Dec 17;11(4):e51. doi: 10.2196/jmir.1258 (PMC2802562; doi:10.2196/jmir.1258)
Supplement: Supplementary file 3 [file jmir_v11i4e51_app3.pdf]

**Department of General Practice, Westmead Hospital**

# Electronic Decision Support Feasibility Study End of study Evaluation Interview guide

THE GEORGE INSTITUTE *for International Health*

---

**Part 1: General overview of the EDS tool**

The aim of the EDS tool is to assist GPs through the provision of decision support for the management of cardiovascular risk. I'd like to start by talking about your personal experience of the EDS tool, and then go on to ask your views about its applicability in general practice more generally.

a. Overall, what do you think was the impact of the EDS tool on the quality of care you were able to provide for your patients?

b. How useful was the EDS tool in supporting communication with your patients?

c. How effective was the EDS tool in assisting you to practise according to national guidelines for cardiovascular risk management?

**Part 2: The EDS output**

I'd now liked to show you some sample printouts from patients enrolled in the study at your practice.

a. What did you find useful about the EDS printout?

b. What information was not helpful in the EDS printout?

c. Was there anything confusing about the printout?

d. How could we improve the printout?

**Part 3: Implementation of the EDS in General Practice**

Our future plans are to integrate the EDS into the commonly used medical software in General Practice.

a. If we do this, what do you see as its potential benefits?

b. Would you anticipate any disadvantages?

c. What barriers do you think we would face? [Probe: practical/ technical/ other]

d. How do you think we could improve it?

e. Would you personally consider using it in your practice? [Probe: why/ why not?]

**Part 4: Wrap up**

a. Are there any other issues not covered that you would like to talk about?
